# Cardiac MRI and CT: the eyes to visualize coronary arterial disease and their effect on the prognosis explained by the Schrödinger's cat paradox

**DOI:** 10.1590/0100-3984.2016.49.1e2

**Published:** 2016

**Authors:** Carlos E. Rochitte

**Affiliations:** 1 Director of Cardiovascular MR and CT Unit at Hospital do Coração (HCor), Associate Professor of Cardiology, Cardiovascular MR and CT Sector, Heart Institute, InCor - Hospital das Clínicas - Univerisity of São Paulo Medical School (InCor/HC-FMUSP), São Paulo, SP Brazil. E-mail: rochitte@incor.usp.br/rochitte@gmail.com/www.rochitte.com.

In the present issue of **Radiologia Brasileira**, Assunção et al.^(1)^ provide a detailed, accurate and updated description of cardiac MRI and CT techniques and their applications in cardiological diseases, more specifically in coronary artery disease, whose clinical relevance does not need to be highlighted. Two recent articles published in this journal have approached relevant aspects of the imaging study of the heart^(2,3)^ as well.

On the grounds of an extensive literature review, the authors describe how the current scientific developments and an appropriately conducted research have led to the present level of utilization, thus benefiting patients in the daily clinical routine, and how under some circumstances such imaging methods are still underutilized.

The following points will be discussed: the scientific development and respective mechanisms, and the utilization of so accurate diagnostic methods.

We must congratulate the authors on their initiative and on the production of that extremely educative text. Also, we should highlight the role played by the authors' institution of origin -Universidade Federal Fluminense - for continually supporting meritocracy and the most recognized researchers at both national and international levels. Even at difficult times like these Brazil is currently experiencing, one can find a silver lining ahead with institutions and individuals appreciating talented people involved in medical research that is not given the due consideration in our country. Such individuals are the ones who will emerge from the dark as true leaders of a future, active and engaged scientific community that will be the driving force for the development of our country.

Similar efforts constitute common examples in the international scientific community. Fortunately, in some rare opportunities, Brazil almost always has a significant participation in such scientific cooperation. The CORE64^(4)^ e C0RE320^(5)^ studies represent personal examples as landmarks or seminal studies, validating coronary CT angiography and myocardial CT perfusion techniques, respectively. Such studies developed under the leadership of Dr. João Lima, from Johns Hopkins University, counted on the personal participation of this author and of the Heart Institute (InCor), Univerisity of São Paulo Medical School, whose patients represented approximately one third of both studies total included patients. The mentioned multicenter studies involved up to 16 countries and were published in recognized and prestigious international scientific journals - **The New England Journal of Medicine** and ** European Heart Journal**, respectively. In addition to the obvious and significant scientific gains which have internationally projected the name of the institution in this area, the research process has allowed for the aquisition of advanced cardiac CT scanners which currently are the institution's structure for carrying on its clinical activities. As Americans say, a true "win-win situation", reflecting, in the research, the model that still nowadays allows for the InCor operation as an island of excellence (assistance to private and health plan patients in order to allow for providing a better assistance to SUS patients and those who do not count on health plan coverage). In our opinion, such a model of international cooperation should be encouraged and appreciated, together with the participation of skilled researchers selected on the basis of meritocracy. Brazilian institutions seem to be starting this type of collaboration that is highly effective and productive for the clinical and preclinical research.

As regards the benefits and use of diagnostic methods in cardiology and general medicine, we are currently following the principles of "Choosing Wisely", "Less is More", and a series of other actions aimed at reasonably trying to restrain the exaggerated use of therapeutic, diagnostic and, particularly, imaging techniques in clinical situations where the benefits to the patient are not proved. We completely agree with such actions and even participate in some of them, principally because the resources on healthcare are restricted and, in fact, very limited for the huge needs of the general population in terms of health assistance. However, it is important to observe that, in the clinical diagnostic practice, the opposite situation is not infrequent, and patients only are submitted to diagnostic tests at advanced stages of disease, already with sequelae and severe complications which if otherwise had been early diagnosed, could be treated, potentially avoiding adverse, many times irreversible outcomes from advanced disease.

A key issue is the lack of discussion about the medical responsibility under the ethical and legal point of view in cases of missed diagnoses and respective consequences which, in cardiology, may be even the patient's death. Particularly, for the emergency physician or for the cardiologist, in many situations of chest pain, critical diagnoses that could only be achieved with imaging methods are imprescindible.

According to some physicians and lawyers involved in such a discussion, a reduction in tests, even those considered to be less necessary, shall be followed by an increase in the risk of missing the diagnosis. As an example, the American College of Radiology recommends that imaging studies are not performed in patients without suspicion of moderate to high pretest probability of pulmonary thromboembolism. Two percent of low-risk patients have pulmonary embolism whose diagnosis will be missed. Severe legal suits may result from such missed diagnoses. Lawyers would tell physicians that, in case anything goes wrong with their patients, nobody will thank them for saving resources of health by not requesting that diagnostic test.

I believe that, in the indication of diagnostic studies, the dialogue with the patient to decide if a determined test is really necessary is the most relevant point in this discussion, together with a careful approach to be adopted on the basis of such diagnostic test results. Many of the situations involving excessive treatment result from an incomplete or erroneous understanding of the diagnostic tests results, particularly from those tests technologically more complex and recently introduced into the clinical practice, such as cardiac MRI and CT.

Finally, my personal opinion is that, in coronary artery disease, the knowledge and observation, by both the physician an the patient, allowed by cardiac MRI and CT, and extremely well demonstrated by Assunção et al.^(1)^, has an independent effect (that is difficult to explain) on the disease course and outcome.

The quantum theory deals with the matter (light) duality behaving as both particle and wave. In the atom particles microcosms, the simple fact that observing a phenomenon changes the phenomenon itself has caused a great debate about the famous theoretical experiment of the Schrödinger's cat^(6)^, where a cat is placed in a box with a vial of poison that would be released by the emission of a single quantum/photon of radioactive material, whose probability is impossible to predict with certainty, thus generating a paradox where before the box is opened, the cat is simultaneously alive and dead. The idea that the chance of the cat being either alive or dead is 50%/50% is inaccurate, uncertain and, in fact, the Schrödinger equation^(6)^ with quotes from Paul Dirac can calculate how much alive and how much dead the cat is. However, as we observe the phenomenon by opening the box, the two waves (alive and dead) collapse into a single wave resulting in an either alive or dead cat.

Already apologizing for the quantum/philosophical digression - in medicine, the observation of the phenomenon itself carries a relevant therapeutic value. Maybe this is just the effect from the partnership (duality) between the physician and the patient, that requires a fact, an image - something palpable, to develop around it, and that does not occur around a probability of disease (that, in general, is vague) and that, in the minds of both the physician and the patient involved in such a relationship, is not something actual and palpable. An optimum physician-patient partnership is, indeed, undoubtedly a major factor of success not only in the disease diagnosis and treatment, but also in the management and promotion of health.
